# Study on the impact of industrial intelligence and the digital economy on China’s regional total factor carbon productivity under carbon neutrality

**DOI:** 10.1038/s41598-026-45039-6

**Published:** 2026-03-20

**Authors:** Dandan Xiao, Jinwang Liu

**Affiliations:** 1 Business School, Shandong University of Political Science and Law, Jinan, 250014 Shandong China; 2 Shandong Provincial Institute of Industry and Information Technology, Jinan, 250014 Shandong China

**Keywords:** Total factor carbon productivity, Global climate change, Green development, Digital economy, Industrial intelligence, Environmental social sciences, Geography, Geography

## Abstract

Improving total factor carbon productivity (TFCP) is the core pathway to China’s low-carbon economic transformation and achieving the “dual carbon” goals. Based on panel data of 30 Chinese provincial-level regions from 2010 to 2023, this paper measures regional TFCP via an undesirable-output super-efficiency SBM model and empirically analyzes the impacts and spatial spillover characteristics of industrial intelligence and the digital economy on TFCP using a Spatial Durbin Model (SDM). Results show China’s TFCP rose overall but exhibited a widening regional gap of “higher in the east, lower in the west”, with significant positive spatial autocorrelation in regional TFCP. The digital economy exerts a significantly positive direct effect and strong positive spatial spillover effect on TFCP, forming a “local driving + spatial radiation” promotion pattern. Industrial intelligence has an insignificantly negative direct effect on local TFCP, yet its positive spatial spillover effect is significant at the 1% level, leading to a significantly positive total effect that reflects its obvious spatial externality, with low-carbon dividends more prominent in regional coordination. Both factors show notable regional heterogeneity: industrial intelligence has a significantly negative direct effect in the east, significantly positive in the central region and insignificant in the west, with positive indirect effects in the east and west; the digital economy presents “local-spillover dual drive” in the east, “local-dominated drive” in the central region and “spillover-dominated drive” in the west. Among control variables, coal-based energy consumption structure and secondary industry-dominated industrial structure significantly inhibit regional TFCP with strong negative spatial spillovers; green finance has an insignificant positive effect, while FDI shows an insignificantly positive direct effect and significantly negative indirect effect due to the “pollution haven” effect. The work clarifies the spatial effects and regional heterogeneity of industrial intelligence and the digital economy on TFCP, providing empirical evidence and policy references for formulating differentiated regional coordination policies, leveraging the two as a “dual engine” to boost China’s regional TFCP and advance high-quality green and low-carbon economic development.

## Introduction

Global climate change is a serious problem for the human society^1, 2, 3^. As a responsible major power, China has explicitly put forward carbon neutrality as the “dual carbon” strategic goals. This commitment is not only a solemn promise to cope with the global environmental crisis but also an inherent requirement and important driving force to achieve high-speed growth towards high-quality growth. Against this backdrop, cities - as concentration areas of energy consumption and carbon emissions - play a pivotal role. The success of low-carbon transformation has a direct effect on the overall success for the national “dual carbon” strategy^4^. Traditionally, energy conservation and emissions reduction have frequently been based on end-of-pipe treatment and mandatory shutdowns, which are costly and may have a negative impact on economic growth. Therefore, how to ensure effective carbon emission reductions without compromising economic growth, i.e., how to ensure synergistic progress between “green” and “development” have become a focal point for academia and policy makers^5, 6^.

Against this background, “TFCP” has arisen. It is defined as the ratio of economic output to carbon dioxide emissions, which takes into account all production factors including capital, labor and energy. Compared with traditional single-factor carbon productivity measures (such as carbon emissions per unit of GDP), TFCP is a more scientific and comprehensive measure of a region’s capacity to balance economic growth with carbon reduction, and its technological efficiency. It is a fundamental indicator to assess the degree of green good development^7^. Enhancing TFCP means that more economic worth is created with fewer carbon emissions through technological advancement and improvement in efficiency^8^. This is the major pathway to achieving a win-win result for both the “dual carbon” goals and economic growth.

So, what is the basic force that is driving the improvement of TFCP? The answer points to industrial intelligence and the digital economy. On one hand, industrial intelligence (represented by artificial intelligence, industrial internet and robotics) is deeply changing the model of manufacturing production. By enabling accurate control of production processes, on-demand energy resource allocation, and predictive maintenance of equipment, it achieves consumption reduction and the whole operation process, and the technological foundation for deep industrial emissions reduction^9^. On the other hand, the digital economy is a new economic paradigm. By permeating, empowering and restructuring data elements, it has given rise to new business models. This reduces societal costs to search for information, match and transaction, fosters optimal resource allocation and lightens and upgrades industrial structures, exerting systemic impacts on carbon emissions^10, 11^.

It is noteworthy that industrial intelligence and the digital economy are not isolated from each other but deeply integrated and mutually reinforcing. Advance in industrial intelligence is based on the data and computing power of the digital economy, and the realization of the value of the digital economy needs to be implemented in real-world economic scenarios such as industrial intelligence. Together, they constitute the “dual engines” of the low-carbon transformation. However, at the theoretical level, how industrial intelligence and the digital economy affect regional TFCP? Do they have spatial spillover effects? At the practical level, despite intensive policy rollouts by governments at all levels, academia has not yet built a clear, unified understanding of the relative importance, pathways and boundary conditions of these two forces in enhancing TFCP. Existing research either focuses on the environmental impact of industrial intelligence or the carbon reduction function of the digital economy. Comprehensive studies which systematically analyze their combined impact on TFCP within the same analytical framework remain scarce.

**Fig. 1:**
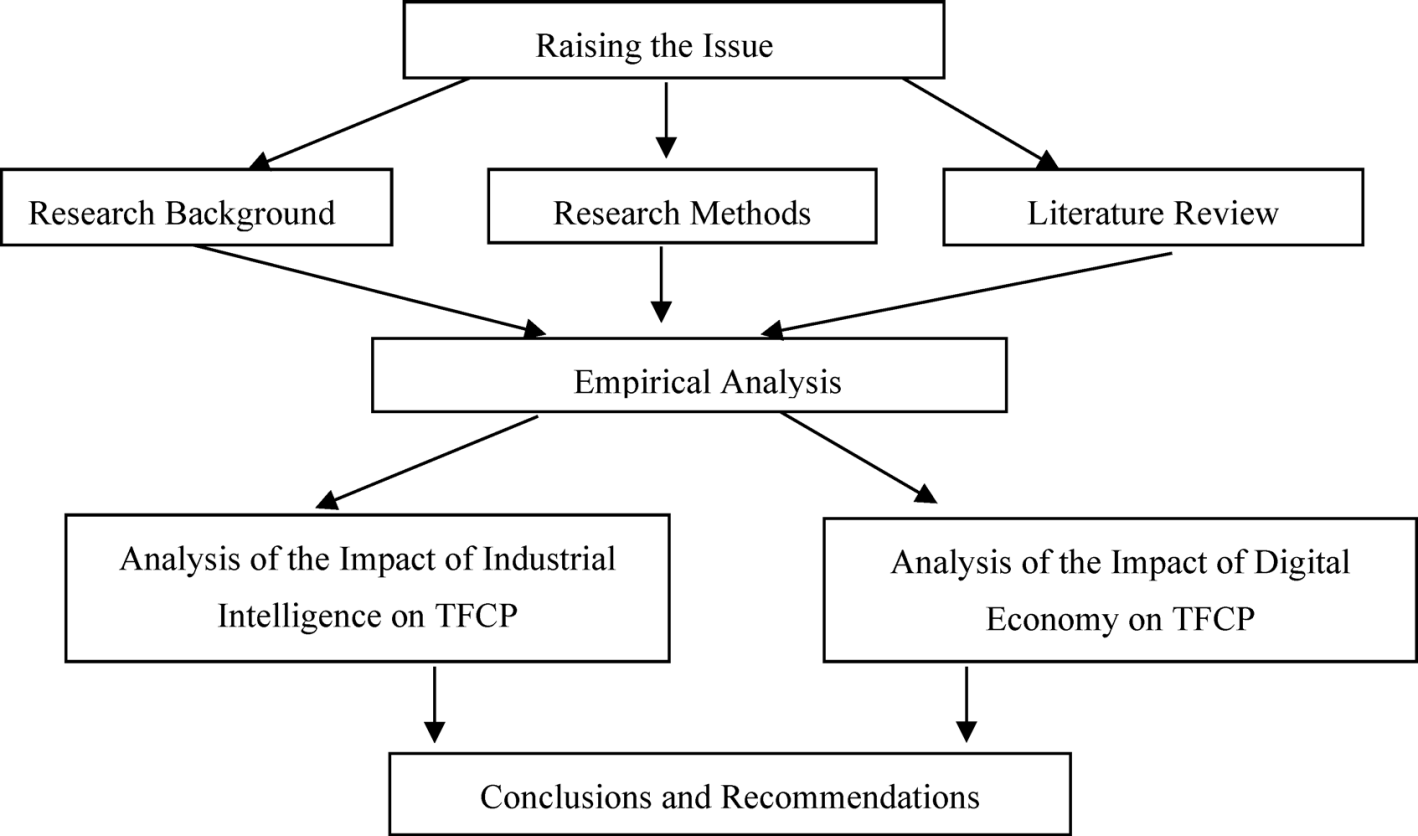
Research framework.

Against this background, the main research questions of this study are as follows: How does industrial intelligence and the digital economy affect regional TFCP in the new stage of development? What are their underpinning mechanisms? Do they have spatial effects? In-depth exploration of such questions not only has great theoretical value, but also serves as important decision-making references for various cities in China on how to implement policies with precision and synergistically promote intelligent transformation and digital development under the dual goals of carbon. The research framework as Fig. 1.

The main contribution is that for the first time, combining the dual driving factors of industrial intelligence and the digital economy into a unified analytical framework to systematically analyze the overall impact of the two factors on TFCP and its spatial mechanism. Empirical results show the existence of substantial spatial spillover effects and regional heterogeneity in their impact on carbon productivity, while explaining their different mechanisms of action. The findings help to lay important theoretical foundations and empirical evidence for regions to design differentiated, coordinated and targeted policies. These policies strive to synergically promote intelligent transformation and digital development to improve the carbon productivity.

## Literature review

The research trajectory of TFCP clearly demonstrates the evolution of the field from conceptual definition to examination of the driving factors. The idea for TFCP came from a goal of a “low-carbon economy.” Early studies mostly used single-factor carbon productivity^12^. However, this approach does not account for the complex effects of substitution among other factors of production and technological progress. Consequently, scholars relied on the principles of TFCP defined as the highest possible ratio of desirable output (e.g. GDP) to undesirable output (CO2) given fixed inputs of production factors (capital, labor, energy). In terms of measurement methodology, mainstream research is based on Data Envelopment Analysis (DEA) models, moving from limited radial and angular models to more sophisticated non-radial, non-angular SBM models^13^, and to the combination of directional distance functions that are able to dynamically decompose growth rates with GML indices^14^. These methods offer sound instruments of empirical research. As for the influencing factors, scholars have identified several traditional drivers such as technology and innovation (e.g. green technology), economic structure (industrial upgrading), government regulation (dual effects of environmental regulations), openness to foreign investment (complex impacts of FDI) as well as urbanization and energy structure^15, 16, 17^. With technological revolution running deep, emerging driving forces such as industrial intelligence and the digital economy are becoming the focus of cutting-edge research.

Regarding the environmental and TFCP impacts of industrial intelligence, a consensus has been reached, where two opposite points of view prevail. Industrial intelligence essentially is the deep integration of next generation information technology and advanced manufacturing technology. Scholars advocating the “emission reduction promotion theory” believe that its net environmental effects are positive, which improves carbon productivity through multiple mechanisms: First, the production optimization effects, reducing waste through real-time monitoring and precise control^18^; second, the intelligent operation and maintenance effects, stabilizing and improving energy efficiency through predictive maintenance; third, the resource allocation effects, optimizing the matching of resources within enterprises and across supply chains through intelligent algorithms. However, another school of research emphasizes the possible “rebound effect” in which efficiency improvements can lead to increased production scales or changes in consumption patterns to other high-energy consuming products^19^, which may partially or fully offset initial energy savings. This debate indicates that industrial intelligence impact on TFCP may not be a simple linear promotion pattern, but its ultimate outcome depends on the relative strength of rebound vs. optimization effects.

The effect of the digital economy on TFCP shows a more systematic and macro level picture. As a new economic model in which data plays an important role as a production factor, its carbon reduction paths are mainly divided into three: First, the technology-empowerment path, in which digital technologies are directly applied to high-energy-consuming industries to achieve intelligent and low-carbon transformation^20^; second, the path of structural optimization, which drives the restructuring of industries toward “servitization” by promoting new business models; third, the path of efficiency enhancement, which takes advantage of the characteristics of data to break down the information barriers and improve the efficiency of resource allocation in the society^21^.However, while recognizing its positive contributions, scholars also recognize the digital economy’s inherent “carbon footprint”, i.e. the energy consumption associated with data centers, network infrastructure and related facilities^22^. This implies that the digital economy may have a “tipping point” in which the impact on carbon productivity is dominated by its own negative carbon footprint, in its early development phase. Only when it starts to empower larger emissions reductions in society, does its positive influence become dominant. This implies there may be an inflection point in the digital economy’s impact on carbon productivity: in its early stages, the negative impacts of its own carbon footprint may be dominant. Only if its positive impacts in making society-wide emissions cuts outweigh its own energy use will it be a net positive on TFCP.

When taking a look at the integration of industrial intelligence and digital economy, we can see that they are not independent entities, but have deep synergistic relationships. Industrial intelligence is the fundamental expression and application scenario of the digital economy in the real economy, and digital economy is the data, computing power, and network infrastructure of industrial intelligence^23^. Their integrated interaction has the following characteristics: Advancements in industrial intelligence will produce a large amount of data, making the digital economy rich in material; on the other hand, the digital economy’s development, through the use of advanced algorithms and powerful computer processing capabilities, will in turn feed back into the industrial intelligence field, driving it to more advanced levels of evolution. A systematic review of existing literature reveals three main shortcomings and research entry points in this field: First, research views are still relatively fragmented, with the majority of studies investigating the two as independent variables while ignoring their intrinsic interconnections and their synergistic effects. Second, mechanism testing is still inadequate with empirical studies systematically identifying and comparing multiple mediating pathways still scarce. Finally, not enough attention is devoted to nonlinear relationships and spatial effects that need to be explored further with regard to their threshold characteristics and spatial spillover effects. Therefore, future research should take an integrated approach, at the same time adding industrial intelligence and the digital economy into the analytical framework. This approach will examine their direct impacts, interactive impacts and the underlying mechanisms on regional TFCP systemically, and further investigate their nonlinear properties and spatial spillover effects. Such research will offer more comprehensive and precise policy insights for understanding and advancing China’s regional green and low carbon transformation.

## Materials and methods

### TFCP measuring

Accurate measurement of TFCP forms the foundation of this study. Traditional carbon emission efficiency assessments often rely on single-factor indicators like carbon intensity, which fail to capture the synergistic inputs and substitution relationships among multiple factors such as capital, labor, and energy. While data envelopment analysis (DEA) models can handle multiple inputs and outputs, traditional radial models (e.g., CCR, BCC) fail to adequately address input-output slackness and cannot further distinguish efficient decision units (those with an efficiency value of 1) on the frontier. To overcome these limitations, this study employs super-efficiency SBM model proposed by Tone^24^. This model further integrates the concepts of undesirable outputs and super-efficiency, rendering it particularly suitable for environmental efficiency assessment.

#### Theoretical foundation of the model

Assume there are n decision-making units (DMUs), each utilizing m inputs $\:x\in\:{R}^{m}$, produce s₁ desirable outputs $\:{y}^{g}\in\:{R}^{{s}_{1}}$ and s₂ undesirable outputs $\:{y}^{b}\in\:{R}^{{s}_{2}}$. Considering the realities of environmental management, this study adopts the assumptions of weak disposability and joint production, meaning that reducing disutility outputs (e.g., carbon emissions) requires sacrificing some resources, which may consequently affect the growth of utility outputs. The production possibility set for the super-efficient SBM model incorporating disutility outputs can be defined as $\:P=\left\{\left(x,{y}^{g},{y}^{b}\right)|x\:can\:product\left({y}^{g},{y}^{b}\right)\right\}$.

For the decision unit DMU_k_ (k = 1,2,…,n) under evaluation, its super-efficient SBM model can be represented as the following linear programming problem:

1 $$\:{\rho\:}^{*}=min\frac{\frac{1}{m}\sum\:_{i=1}^{m}\:\frac{{\stackrel{-}{x}}_{i}}{{x}_{ik}}}{\frac{1}{{s}_{1}+{s}_{2}}\left(\sum\:_{r=1}^{{s}_{1}}\:\frac{{\stackrel{-}{y}}_{r}^{g}}{{y}_{rk}^{g}}+\sum\:_{q=1}^{{s}_{2}}\:\frac{{\stackrel{-}{y}}_{q}^{b}}{{y}_{qk}^{b}}\right)}$$

2 $$s.t.\left\{ {\begin{array}{*{20}c} {\bar{x} \ge \mathop \sum \limits_{{j = 1,j \ne k}}^{n} \lambda _{j} x_{j} ,\bar{y}^{g} \le \mathop \sum \limits_{{j = 1,j \ne k}}^{n} \lambda _{j} y_{j}^{g} ,\bar{y}^{b} \ge \mathop \sum \limits_{{j = 1,j \ne k}}^{n} \lambda _{j} y_{j}^{b} } \\ {\bar{x} \ge x_{k} ,\bar{y}^{g} \le y_{k}^{g} ,\bar{y}^{b} \ge y_{k}^{b} } \\ {\lambda _{j} \ge 0,j = 1,2,...,n\left( {j \ne k} \right)} \\ \end{array} } \right.$$

Where: $\:{\rho\:}^{*}$ represents the TFCP value being sought. When $\:{\rho\:}^{*}$ ≥ 1, it indicates that the DMU lies on or above the production frontier surface and is efficient, with higher values signifying greater efficiency. When $\:{\rho\:}^{*}$ < 1, it indicates that the DMU is inefficient. $\:{\lambda\:}_{j}$ is the intensity variable used to construct the best practice frontier surface. $\:\stackrel{\prime }{x},{\stackrel{\prime }{y}}^{g},{\stackrel{\prime }{y}}^{b}$ represent the projected values of DMU_k_ onto the frontier surface, signifying the target values for achieving an efficient state.

#### Variable selection and data description

Based on principles of scientific rigor and data availability, this study constructs the following input-output indicator system (Table 1).

Capital Input: Following Bai and Sun^25^, regional fixed capital stock is selected as the key indicator, with the specific formula being:$\:{K}_{t}={I}_{t}+(1-\delta\:){K}_{t-1}$, where $\:{I}_{t}$ represents the current year’s fixed asset investment, δ denotes the depreciation rate, This paper adopts Shan^26^ depreciation rate of 5 and 10.96%, and selects 2010 as the base period when setting the base period.

Labor Input: The number of employed persons per unit area at year-end serves as the metric for labor input^27^. This indicator provides a direct reflection of a city’s labor force size and constitutes a crucial basis for evaluating labor input conditions.

Energy Input: Multiple energy sources are utilized in social production processes. However, due to differing statistical units for various energy types, the total energy input must be converted into a unified unit. Following existing research, eight energy sources including coal, coke, and crude oil are converted into standard coal equivalents^28^.

Expected Output: Measured using actual GDP indicators for each region^29^, with 2010 as the base year. GDP deflators for each city were applied to eliminate price effects.

Non-Expected Output: Measured using carbon dioxide emissions for each region^30^. Since carbon emissions primarily stem from fossil fuel consumption, this study categorizes fossil fuels into eight types based on the classification standards of the China Energy Statistical Yearbook. When calculating non-expected output, the method recommended by the IPCC is adopted, with the specific formula as follows:

3 $$\:{CO}_{2}=\sum\:_{i=1}^{8}{\left({CO}_{2}\right)}_{i}=\sum\:_{i=1}^{8}{E}_{i}\times\:{NCV}_{i}\times\:{CEF}_{i}\times\:{COF}_{i}\times\:\frac{44}{12}\:$$

CO₂ emissions are determined by the consumption of various fossil fuels(E), their average lower heating value (NCV), carbon emission factors (CEF), and carbon oxidation rates (COF).

**Table 1:** Input-output indicator system.

Indicator	Category	Indicator composition	Indicator measurement
Input indicators	Elemental inputs	Labor force	The total number of employees in the region
		Capital	Regional fixed capital stock
		Energy	Total energy consumption
Output indicators	Desired output	Actual regional gross domestic product	Real regional GDP (in billions of yuan) adjusted for price factors using the GDP deflator, with 2010 as the base year
	Non-desired outputs	Total regional carbon dioxide emissions	Following the methodology recommended by the IPCC, estimates are based on the consumption levels of major energy sources such as coal, gasoline, diesel, and natural gas.

### Spatial econometric models

#### Spatial autocorrelation test

Before constructing spatial econometric models, it needs to first test whether the research object (i.e., TFCP) exhibits spatial autocorrelation. If no significant spatial dependence is found, ordinary panel models suffice without requiring more complex spatial econometric methods. This study employs a global spatial autocorrelation test. The global Moran’s I index serves as a classic metric for assessing spatial clustering patterns of attribute values across the entire study area. Its calculation formula is:

4 $$\:I=\frac{n}{{S}_{0}}\times\:\frac{\sum\:_{i=1}^{n}\sum\:_{j=1}^{n}{w}_{ij}({x}_{i}-\stackrel{\prime }{x})({x}_{j}-\stackrel{\prime }{x})}{\sum\:_{i=1}^{n}({x}_{i}-\stackrel{\prime }{x}{)}^{2}}$$

Where: I denotes the global Moran’s I index value. n represents the total number of regions. $\:{x}_{i}$ and $\:{x}_{j}$denote the TFCP of region i and region j, respectively. $\:\stackrel{\prime }{x}$ is the average TFCP across all regions. $\:{w}_{ij}$ is an element of the spatial weight matrix W. $\:{S}_{0}={\sum\:}_{i=1}^{n}{\sum\:}_{j=1}^{n}{w}_{ij}$ is the sum of all elements in the spatial weight matrix.

#### Spatial econometric model specification

The First Law of Geography states that all phenomena exhibit spatial relationships, with proximity enhancing correlation. Economic activities (e.g., technology diffusion, industrial relocation) and environmental pollution (e.g., transboundary air pollutant transport) demonstrate pronounced spatial dependence. Ignoring this dependence introduces estimation bias. Therefore, the work employs spatial econometric methods to examine whether significant spatial spillover effects exist between industrial intelligence and the digital economy.

#### Construction of the spatial weight matrix

The spatial weight matrix *W* is central to spatial econometric models, quantitatively defining spatial relationships between units. This study constructs Inverse Distance Weighted Matrix: =1/ (≠), where is the geographic distance between the government seats of the two regions. This matrix assumes spatial interaction intensity diminishes with distance. All matrices undergo row normalization prior to modeling.

#### Model specification and selection

Against the background of economic integration of the world and regional cohesion, new economic geography focuses on the spatial dimension that was neglected in traditional economics and emphasizes that economic units do not exist in isolation but are inherently related to their neighbors. The shorter the distance between economic units, the more the surrounding regions will influence their economic development. Incorporating spatial factors when analyzing the contribution of economic variables in improving TFCP enables a more accurate reflection of the complexity in the real world. This study uses the spatial econometric models to analyze the relationship between digital economy and industrial intelligence on TFCP. The three conventional spatial econometric models, namely spatial autoregression (SAR), spatial error model (SEM), and spatial Durbin model (SDM) are used. In order to choose the most appropriate model for this research, we successively performed the Lagrange multiplier test (LM test), likelihood ratio test (LR test), and Wald test. These tests provide for the scientific selection of an appropriate model. The results of the test show the SDM model is chosen for analysis (Table 2).

**Table 2:** Spatial econometric model selection tests.

Testing method	Statistic	Testing method	Statistic
LM (lag) test	110.187 ***	lr_both_ind	154.24 ***
Robust LM (lag) test	22.038 ***	lr_both_time	301.64 ***
LM (error) Moran	11.254 ***	Wald_spatial_lag	64.09 ***
LM (error) test	117.474 ***	LR_spatial_lag	60.28 ***
Robust LM (error) test	29.325 ***	Wald_spatial_error	53.98 ***
Hausman test	24.11 ***	LR_spatial_error	50.56 ***

The general expression for the spatial Dubein model is:

5 $$\begin{aligned} TFC{P_{it}} & =\rho W \cdot TFC{P_{jt}}+{\beta _1}D{E_{it}}+{\beta _2}I{I_{it}}+{\theta _1}W \cdot D{E_{jt}}+{\theta _2}W \cdot I{I_{jt}} \\ & +\gamma {X_{it}}+{\mu _i}+{\lambda _t}+{\varepsilon _{it}} \\ \end{aligned}$$

Among these, $\:{\mathrm{X}}_{it}$ denotes the vector of control variables. DE represents the digital economy, II denotes industrial intelligence, ρ is the spatial autoregressive coefficient, and $\:{\theta\:}_{1},{\theta\:}_{2}$ are the spatial interaction coefficients of the explanatory variables.

### Variable selection

#### Explained variable

This paper utilizes the super-efficiency SBM introduced earlier to calculate China’s regional TFCP.

#### Dependent variable

Industrial intelligence (II). Existing research lacks a universally accepted metric for industrial intelligence. Our review identifies two predominant approaches: single-indicator methods and composite indicator methods. Drawing on prior scholarly work^31, 32^, this study search for artificial intelligence enterprises on Tianyancha while retaining active companies, match them with provincial regions, and take the logarithm of the results to obtain the level of industrial intelligence.

Digital Economy (DE). Drawing on previous scholarly research^33, 34^, this paper constructs a digital economy indicator system as shown in Table 3. This system integrates comprehensive measurement frameworks from international organizations (e.g., OECD) and China’s National Bureau of Statistics’ “Statistical Classification of the Digital Economy and Its Core Industries.”

**Table 3:** Digital economy indicator system.

Primary indicator	Secondary indicator	Tertiary indicator	Indicator attribute
Digital infrastructure	Internet penetration rate	Internet broadband access port	+
		Internet broadband access users	+
		Number of domains	+
	Mobile phone penetration rate	Mobile phone call duration	+
		Mobile phone penetration rate	+
Digital industrialization	Software and information technology services	Software business revenue as a percentage of GDP	+
		Urban employment in information transmission, software, and information technology services	+
	Development level of the electronic information manufacturing industry	Information technology services revenue as a percentage of GDP	+
		Telecommunications service volume as a percentage of GDP	+
		Telecommunications service volume per capita	+
	Level of development in the postal and telecommunications industry	Per capita postal service volume	+
		E-commerce sales	+
Industrial digitalization	Level of digital development	Percentage of enterprises engaged in e-commerce transactions	+
		Number of computers per 100 people	+
		Number of websites per 100 enterprises	+
Digital innovation capability	Research and experimental development level	Number of R&D projects in industrial enterprises above designated size	+
	Technological innovation capability	Technology market transaction volume	+
		Number of domestic patent applications granted	+

#### Control variables

Green Finance (GF). Some researchers have proposed methods that measure single indicators, for example, choosing credit and green insurance as reference points in determining green finance development. The benefit of this approach is the intuitive ease of use which allows for a rapid outline of green finance development. However, green finance is hard to fully characterize using a single indicator. Consequently, other researchers have adopted a more comprehensive evaluation system, in which different indicators are appropriately weighted in order to obtain a holistic measure. This holistic approach enables the evaluation of green finance development from various angles and dimensions instead of being limited to a single perspective, thus enabling more in-depth and detailed analysis. The entropy method is a measurement of the dispersion of each indicator in the whole evaluation system by calculating the entropy value. This approach excludes any subjective bias in the process of attribution, which objectively determines weight coefficients based on the actual distribution of indicator data only. This guarantees the objectivity and scientific nature of the results of the evaluation. Therefore, this paper selects the entropy method to measure the index of the dynamic development level of green finance in each region. Building on the green finance indicator construction frameworks suggested by He et al.^35^ and Wang et al.^36^, this paper constructs a comprehensive evaluation indicator system. Thereafter, the entropy method is used to measure the indicators in this system. The specific content and definitions of each of the indicators can be found in Table 4.

**Table 4:** Green finance indicator system.

Primary indicator	Secondary indicator	Tertiary indicator
Green finance	Green credit	Total environmental protection project loans / total loans
	Green securities	Total green bond issuance / total bond issuance
	Green insurance	Promotion of environmental pollution liability insurance
	Green investment	Regional environmental pollution control investment / regional GDP
	Green support	Fiscal environmental protection expenditures / fiscal general budget expenditures
	Green fund	Total market value of green funds / total market value of all funds
	Green rights	Total transaction value of energy rights trading, emission rights trading/equity market trading

Industrial Structure (IS). This paper focuses on the share of secondary industry output in regional GDP in order to estimate the effect of industrial structure on TFCP. The digital economy and use of digital technologies promote the optimization of industrial structure, and promote the transformation of traditional manufacturing industries to high-tech industries and service industries. This gives new impetus to improving energy efficiency and pollution.

Energy Consumption Structure (ECS). Adjusting the energy consumption structure can effectively promote the reduction of emissions. This indicator follows the methodology of Shao et al.^37^, in the form of the ratio of coal consumption to total energy consumption. To make statistical results consistent, in order to calculate the terminal consumption of each energy type, the standard coal conversion factors for various energy sources published in the China Energy Statistical Yearbook are used in this study. The last indicator is then in the form of the share of coal terminal consumption in the total energy terminal consumption.

Foreign Direct Investment (FDI) Level. Two main academic views exist with respect to the effect of FDI on regional TFCP: First, the “pollution halo” effect, which states that the inflow of FDI spreads advanced technology and management know-how, which in turn would positively affect the technology of production methods of host countries, and thus improve regional TFCP; Second, the pollution haven hypothesis, which states that FDI may lead to the shifting of high pollution and energy-intensive industries to countries or regions with more relaxed environmental regulations. Such behavior could worsen environmental pollution in the host countries, hence suppressing improvements in regional TFCP. FDI levels are usually measured by the ratio of real foreign capital utilization and GDP at the prefecture-level city level^38^.

All variable data above are sourced from relevant data spanning 2010–2023 across China’s 30 provincial-level regions (including all provincial-level regions and autonomous regions, excluding Hong Kong, Macao, Taiwan, and Tibet). Data sources include the China Statistical Yearbook, China Urban Statistical Yearbook, China Energy Statistical Yearbook, China Industrial Statistical Yearbook, and provincial statistical yearbooks.

## Results

### Subsection

**Fig. 2:**
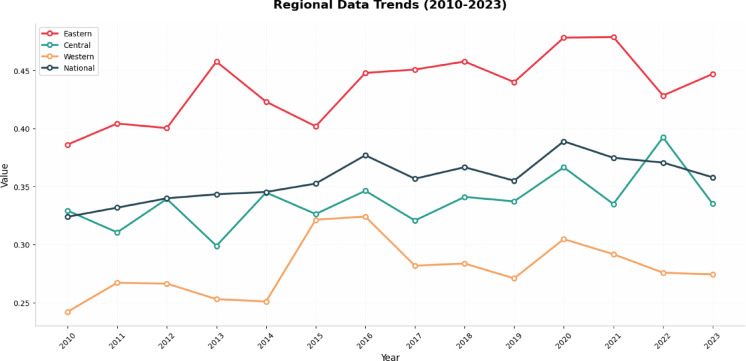
The trends of TFCP in eastern, central, western and national.

The results of measuring TFCP using the super-efficiency SBM model are presented in Table 5 below and Fig. 2 shows the trends in TFCP across national, eastern, central, and western regions.

**Table 5:** Measurement results of TFCP.

Region	Province	2010	2011	2012	2013	2014	2015	2016	2017	2018	2019	2020	2021	2022	2023	Mean
Eastern Region	Beijing	0.3226	0.2374	0.2163	0.2016	0.3922	0.4059	0.2983	0.2873	0.2699	0.3744	0.2466	0.3377	0.2333	0.2202	0.2888
	Tianjin	0.1987	0.3586	0.4185	0.2947	0.2819	0.2726	0.3748	0.2514	0.3510	0.2398	0.2467	0.2072	0.3587	0.4506	0.3075
	Hebei	0.3028	0.2974	0.2821	0.3871	0.2562	0.3865	0.2566	0.2546	0.2050	0.3634	0.3403	0.3223	0.3188	0.2817	0.3039
	Liaoning	0.3312	0.2607	0.4109	0.2761	0.2684	0.2200	0.3812	0.3517	0.3385	0.3363	0.3052	0.3494	0.2710	0.4382	0.3242
	Shanghai	0.2947	0.2870	0.2390	0.4123	0.3872	0.3635	0.3624	0.3271	0.3772	0.2875	0.4794	0.3225	0.2476	0.2599	0.3320
	Jiangsu	0.4517	0.4308	0.3914	0.3894	0.3483	0.3984	0.3031	0.5035	0.3515	0.2915	0.2852	0.5024	0.4758	0.4262	0.3964
	Zhejiang	0.4152	0.3736	0.4225	0.3196	0.5359	0.3702	0.3039	0.2611	0.5561	0.5351	0.4853	0.4364	0.4026	0.4408	0.4184
	Fujian	0.3366	0.5854	0.3751	0.3141	0.2710	0.5283	0.6437	0.5677	0.5313	0.4395	0.6736	0.3469	0.6382	0.3989	0.4750
	Shandong	0.3311	0.2765	0.6299	0.6607	0.5515	0.5699	0.4190	0.6651	0.3447	0.7265	0.4258	0.3580	0.2993	0.6679	0.4947
	Guangdong	0.7637	0.6050	0.6394	0.4515	0.6978	0.3661	1.0222	0.4499	0.3857	0.3145	0.7006	1.2209	0.6152	0.7228	0.6397
	Hainan	0.4969	0.7323	0.3775	1.3247	0.6616	0.5373	0.5591	1.0373	1.3213	0.9295	1.0691	0.8610	0.8502	0.6082	0.8119
Eastern Region Average		0.3860	0.4041	0.4002	0.4574	0.4229	0.4017	0.4477	0.4506	0.4575	0.4398	0.4780	0.4786	0.4282	0.4468	0.4357
Central Region	Shanxi	0.1613	0.1792	0.1872	0.1641	0.3016	0.2858	0.2540	0.2632	0.2645	0.2303	0.1637	0.1860	0.1947	0.1736	0.2149
	Inner Mongolia	0.3089	0.2827	0.2553	0.2640	0.2712	0.2338	0.1657	0.1944	0.2046	0.1819	0.3200	0.2867	0.2671	0.2809	0.2512
	Jilin	0.2899	0.2417	0.1692	0.2059	0.2003	0.1969	0.3093	0.2831	0.2505	0.2853	0.3126	0.2400	0.1770	0.2202	0.2416
	Heilongjiang	0.2143	0.2099	0.3308	0.2955	0.2622	0.3035	0.3394	0.2558	0.1852	0.2368	0.2448	0.2299	0.3579	0.3102	0.2697
	Anhui	0.2820	0.3305	0.3835	0.2710	0.1941	0.2545	0.2640	0.2426	0.3814	0.3358	0.3011	0.3506	0.4263	0.2879	0.3075
	Jiangxi	0.2063	0.2715	0.2867	0.2599	0.4091	0.3650	0.3276	0.3845	0.4730	0.3076	0.2191	0.2638	0.3167	0.2796	0.3122
	Henan	0.4014	0.4117	0.3874	0.4283	0.5317	0.3320	0.2256	0.2468	0.3267	0.3145	0.4300	0.4308	0.4178	0.4539	0.3813
	Hubei	0.5450	0.3365	0.2325	0.2696	0.3516	0.3389	0.4428	0.4467	0.4191	0.4426	0.5670	0.3170	0.2510	0.2897	0.3750
	Hunan	0.3769	0.3376	0.4703	0.4840	0.4693	0.4796	0.6134	0.3291	0.2623	0.3008	0.3859	0.3597	0.4735	0.5143	0.4183
	Guangxi	0.5051	0.5030	0.6893	0.3456	0.4556	0.4725	0.5203	0.5595	0.6416	0.7349	0.7204	0.6832	1.0401	0.5399	0.6008
Central Region Average		0.3291	0.3104	0.3392	0.2988	0.3447	0.3262	0.3462	0.3206	0.3409	0.3371	0.3664	0.3348	0.3922	0.3350	0.3373
Western Region	Chongqing	0.2553	0.2566	0.1553	0.2085	0.2338	0.1644	0.1224	0.1364	0.1638	0.2453	0.2706	0.1629	0.2138	0.2409	0.2021
	Sichuan	0.1732	0.1200	0.1405	0.1616	0.2645	0.2878	0.1646	0.2125	0.2535	0.1793	0.1244	0.1415	0.1622	0.2814	0.1905
	Guizhou	0.2667	0.1827	0.2235	0.2428	0.1735	0.1281	0.1448	0.1611	0.2974	0.2933	0.1933	0.2389	0.2561	0.1805	0.2130
	Yunnan	0.1362	0.1510	0.1653	0.3389	0.3265	0.2113	0.2596	0.2720	0.1934	0.1438	0.1572	0.1738	0.3713	0.3520	0.2323
	Shaanxi	0.2270	0.2767	0.2885	0.2194	0.1531	0.1696	0.1812	0.4081	0.3771	0.2437	0.2973	0.3076	0.2123	0.1619	0.2517
	Gansu	0.1725	0.1836	0.4524	0.4202	0.2666	0.3174	0.3354	0.2280	0.1717	0.1853	0.2053	0.5082	0.4621	0.2770	0.2990
	Qinghai	0.3769	0.3401	0.2395	0.1817	0.1894	0.2090	0.5790	0.4822	0.2782	0.3921	0.3501	0.2402	0.1862	0.1945	0.3028
	Ningxia	0.2119	0.6284	0.5367	0.2955	0.4192	0.3705	0.2532	0.1891	0.2034	0.2233	0.6867	0.5686	0.3064	0.4387	0.3808
	Xinjiang	0.3583	0.2643	0.1951	0.2076	0.2313	1.0339	0.8763	0.4462	0.6140	0.5311	0.4565	0.2824	0.3110	0.3406	0.4392
Western Region Average		0.2420	0.2670	0.2663	0.2529	0.2509	0.3213	0.3240	0.2817	0.2836	0.2708	0.3046	0.2916	0.2757	0.2742	0.2790
National average		0.3238	0.3317	0.3397	0.3432	0.3452	0.3524	0.3768	0.3566	0.3665	0.3549	0.3888	0.3746	0.3705	0.3578	0.3554

Using the aforementioned methodology, TFCP was measured for China’s 30 provinces from 2010 to 2023. The specific results are presented in Table 5 below. As shown in Table 5; Fig. 2, during the period from 2010 to 2023, Hainan, Guangdong, Guangxi, Shandong, Fujian, and Xinjiang ranked among the top provinces in terms of TFCP levels nationwide. Conversely, Chongqing, Sichuan, Guizhou, and Shanxi ranked at the bottom among the 30 provinces. Overall, from 2010 to 2023, as provinces increasingly prioritized environmental protection, the TFCP levels of provinces improved to some extent. The result shows the average TFCP and its fluctuation in the eastern, central and western regions, and the average of the country in the period from 2010 to 2023. It is evident that the eastern region’s average TFCP significantly exceeds the national average, which in turn surpasses the central region’s average. The central region’s average TFCP is higher than that of the western region.

### Spatial autocorrelation test

Prior to spatial econometric regression, it is necessary to establish whether there are spatial autocorrelation characteristics in carbon productivity. The results of the test are presented in Table 6. Although the Moran’s I index for TFCP could fluctuate between 2010 and 2023 during the study period, it was significantly positive all the time. This suggests that the TFCP in all the regions showed significant positive spatial autocorrelation and stable spatial dependence over the period 2010–2023, which justifies the subsequent spatial regression analysis.

**Table 6:** Spatial autocorrelation test results.

Years	Moran’I	*P*-value
2010	0.346	0.000
2011	0.312	0.000
2012	0.293	0.000
2013	0.319	0.000
2014	0.309	0.000
2015	0.295	0.000
2016	0.285	0.000
2017	0.313	0.000
2018	0.318	0.000
2019	0.262	0.000
2020	0.255	0.000
2021	0.247	0.001
2022	0.152	0.017
2023	0.175	0.010

### Spatial estimation results

In the SDM model, the dependent variable is not only affected by the local independent variables but also by independent variables in the neighboring areas. This means that values in an area are not only dependent on the area’s own characteristics but may also be influenced by the surrounding area. Due to this kind of spatial interaction, the assumption of unidirectional causality is not met and the traditional methods of regression are unable to properly capture the effects of space in the data generation process. OLS regression does not provide accurate results. Therefore, in this study, dual fixed effects in the framework of SDM are used for regression estimation to study the spatial spillover effect of digital economy and industrial intelligence on TFCP. The results of the regression estimation are shown in Table 7.

**Table 7:** Benchmark spatial estimation results.

Variables	(1)	(2)
TFCP	Main	Wx
II	-0.0195	0.1348 **
	(-1.08)	(2.59)
DE	0.7983 ***	1.2216 ***
	(9.55)	(5.21)
GF	0.0663	0.4489
	(0.33)	(0.66)
IS	-0.0021 *	-0.0091 **
	(-1.84)	(-2.51)
ECS	-0.1652 **	-1.0974 ***
	(-2.36)	(-4.23)
FDI	0.0539	-1.0385 *
	(0.19)	(-1.72)
Fixed province	YES	
Fixed year	YES	
rho	-0.3518 ***	
	(-3.32)	
Sigma2_e	0.0027 ***	
	(14.19)	
Sample size	420	

The value of spatial autocorrelation coefficient, rho, for TFCP passed the significance test, implying a strong spatial spillover effect of regional TFCP in China. This means that local TFCP alterations have a strong influence on TFCP in other regions. Therefore, regional carbon emission governance should not be limited to each region, and inter-city joint prevention and control measures should be taken to reduce carbon emissions, and inter-regional carbon leakage should be prevented. The analysis further confirms the need to include spatial factors in the TFCP research. Considering the relatively better model fit under fixed-time effects, the Model (2) from Table 7 is chosen for further discussion. Table 7 shows preliminary estimation results of the spatial econometric model, which, despite considering the impact of variables on local and neighboring regions, still has limitations. In order to analyze the spatial spillover effects more precisely, we further use spatial regression partial differential method for in-depth analysis.

### Decomposition of total effects

To further analyze the spatial spillover effects, based on existing research^39, 40, 41^, we decomposed the total effects of the SDM model. This decomposition allows the distinction between direct effects and indirect effects in a region and assists in identifying factors which are driven internally versus from the interactions between regions. As shown in Table 8.

**Table 8:** Decomposition results of total effects.

	(1)	(2)	(3)
	LR_Direct	LR_Indirect	LR_Total
II	-0.0249	0.1091 ***	0.0842 **
	(-1.31)	(2.67)	(2.01)
DE	0.7555 ***	0.7275 ***	1.4830 ***
	(8.79)	(4.36)	(10.70)
GF	0.0666	0.3751	0.4417
	(0.35)	(0.73)	(0.81)
IS	-0.0018	-0.0064 **	-0.0081 ***
	(-1.47)	(-2.20)	(-2.98)
ECS	-0.1189 *	-0.8228 ***	-0.9418 ***
	(-1.74)	(-3.95)	(-4.59)
FDI	0.1134	-0.8127 *	-0.6993
	(0.41)	(-1.72)	(-1.28)

Table 8 presents the spatial-effect decomposition results for the spatial Durbin model. The results show that the direct effect of digital economic development is 0.7555, which means that the development of the digital economy in this region is conducive to the improvement of local TFCP. This is because the energy consumption and carbon emissions in digital economy-related industries are relatively low, mainly because the industries are dominated by internet enterprises and information services. Furthermore, they can change industrial structures marked by high-energy-consumption and high-emission industries and hence lead to regional low-carbon transitions, thereby improving local TFCP. The indirect effect coefficient is also positive, which shows that improvements in the digital economy development level of neighboring regions also promote the increase in the TFCP in this region, indicating that digital economic development has spatial spillover effects. This is due to the fact that the digital economy frees the activities of regions from geographical restrictions on economic operations. The digital economy makes use of the dynamic spatial spillover channels, including the diffusion of knowledge and technology (talent mobility, supply chain collaboration, academic exchange), industrial chain linkage (removing geographical constraints to enable cross-regional industrial division and cooperation), market forces (fostering the integration of regional commodity, service and factor markets), and model demonstration and imitation (changing local consumer demand and preferences and creating demonstration effects). These dynamic spatial spillover channels allow the digital economy to function as a “catalyst” and “connector” for the coordinated green and low-carbon development across regions, which indirectly drives the improvement of the TFCP in neighboring areas. This process contains the essence of its spatial spillover effects.

The direct effect of industrial intelligence on TFCP is negative but not statistically significant. This stems from short-term suppression caused by increased capital investment and energy consumption during the initial phase of intelligent transformation, as well as the time-lag effects of technology adaptation and learning costs. Additionally, regional heterogeneity in industrial foundations weakens its statistical significance. The indirect effect is significantly positive at the 1% level and serves as the core driving force: low-carbon technologies and management expertise from smart factories within a region spill over to neighboring areas through channels such as industrial chains and talent mobility, driving energy efficiency improvements in surrounding enterprises. Simultaneously, industrial intelligence propels regional industries toward cleaner transformation, forming a “center-periphery” green collaborative network. Combined with policy demonstration and standard-driven pressure from highly intelligent regions, this creates significant positive spatial spillovers. The total effect is significantly positive at the 5% level, indicating that the positive spillover intensity of the indirect effect (0.1091) far exceeds the weak negative impact of the direct effect (-0.0249). This reflects the spatial externalities characteristic of industrial smart transformation, where its low-carbon dividends are more evident at the regional coordination level, serving as an effective means to enhance regional TFCP.

The direct, indirect, and total effects of green finance are all insignificant, indicating that green finance development remains in its infancy. Policy implementation and resource allocation face time lags and regional imbalances, failing to establish a stable driving force for *TFCP*. The direct effect of industrial structure is insignificantly negative, while its indirect and total effects are significantly negative at the 5% and 1% level, respectively. This indicates that industrial structure upgrading in this region exerts a relatively weak inhibitory effect on local carbon productivity. However, it may export high-energy-consuming processes to neighboring regions through industrial transfer and demand transmission, thereby intensifying surrounding carbon emission pressures. Ultimately, this manifests as a significant overall inhibition at the regional level, reflecting that the current industrial structure transformation remains extensive in nature, with insufficient green upgrading. The direct effect (significant at 10%), indirect effect, and total effect (both significant at 1%) of energy consumption structure were all negative, with the indirect effect being substantially stronger than the direct effect. This indicates that a fossil-fuel-dominated consumption pattern not only directly elevates local carbon emissions but also generates strong negative spatial spillovers through energy trade and pollution transmission, exerting a powerful comprehensive suppression on regional *TFCP*. This underscores the urgency of optimizing the energy structure. The direct effect of foreign direct investment (FDI) is not significantly positive, while the indirect effect is significantly negative at the 10% level. with the overall effect not significantly negative. This suggests that while FDI may marginally enhance local efficiency, it could potentially shift energy-intensive industries to neighboring regions through a “pollution refuge” effect, squeezing the space for green industries in surrounding areas. Its negative spillover offsets the local positive effects, failing to generate regional green synergies. Overall, energy consumption structure and industrial structure are the core variables suppressing regional *TFCP*, with their negative spatial spillover effects dominating—reflecting pronounced “regional linkage pollution” characteristics. The positive driving roles of green finance and FDI remain underutilized, necessitating optimized spatial allocation to guide their shift toward low-carbon sectors.

### Heterogeneity analysis

There are huge gaps in China between economic policies, endowments of resources and level of technology. As shown by the above measurement results, the TFCP of China also has regional differentiation. Consequently, the influence relationship among these variables will present the characteristics of heterogeneity. Accordingly, this study divides the research sample into three regions namely Eastern, Central and Western based on the official classification standards. Sub-sample regressions are performed to investigate the regional differences of the impact of industrial intelligence and the digital economy on the carbon productivity in China. Empirical analysis uses a 0–1 spatial weighting matrix where the results are shown in Table 9.

**Table 9:** Heterogeneity analysis results.

			II		DE	
Eastern Region	(1)	LR_Direct	-0.1464 ***	(-4.28)	0.6908 ***	(5.13)
	(2)	LR_Indirect	0.1087 *	(1.96)	0.7075 **	(2.58)
	(3)	LR_Total	-0.0378	(-0.84)	1.3983 ***	(5.34)
Central region	(1)	LR_Direct	0.0873 **	(2.34)	0.8035 ***	(3.64)
	(2)	LR_Indirect	-0.0240	(-0.27)	-0.1018	(-0.29)
	(3)	LR_Total	0.0632	(0.57)	0.7017 **	(2.24)
Western region	(1)	LR_Direct	-0.0318	(-0.93)	0.1437	(0.70)
	(2)	LR_Indirect	0.2042 **	(2.55)	1.6849 ***	(2.79)
	(3)	LR_Total	0.1724 *	(1.89)	1.8286 ***	(3.11)

The impact of industrial intelligence and the digital economy on TFCP exhibits significant spatial differentiation. In the eastern region, the direct effect of industrial intelligence is significantly negative at the 1% level (-0.1464***), while the indirect effect is significantly positive at the 10% level (0.1087*). The overall effect is insignificant, reflecting that as a pioneer region in intelligent transformation, the eastern region faces significant local adjustment costs during the initial transition phase. Substantial capital investments in smart equipment upgrades and digital platform construction temporarily divert resources from low-carbon technology R&D. Simultaneously, the automation upgrade phase temporarily increases energy consumption, suppressing local carbon productivity. Meanwhile, the eastern region’s accumulated expertise in smart low-carbon technologies and digital management diffuses to surrounding areas through industrial chain collaboration and talent mobility, generating significant positive spatial spillovers. The counterbalancing effects of these two mechanisms result in the overall effect being insignificant. In contrast, the eastern region’s digital economy exhibits highly significant positive direct effects (0.6908***), indirect effects (0.7075**), and total effects (1.3983***) are all highly significantly positive, revealing a dual-engine model of “local-driven + spatial spillover.” This stems from the eastern region’s robust digital infrastructure and efficient factor mobility, where digital technologies deeply integrate with the real economy while data elements and green digital services permeate surrounding areas, highlighting pronounced regional synergies.

In the central region, the direct effect of industrial intelligence is significantly positive at the 5% level (0.0873**), while the indirect and total effects are insignificant. This aligns with the central region’s mid-stage industrialization phase: Traditional manufacturing holds a high proportion, where intelligent upgrades can rapidly boost production efficiency and optimize energy allocation, directly enhancing local carbon productivity. However, constrained by interregional administrative barriers and insufficient industrial coordination, the cross-regional diffusion mechanism for intelligent technologies remains underdeveloped. Indirect effects are weak, partially offsetting positive direct effects, ultimately resulting in non-significant overall effects. The direct effect of the digital economy in central China is significantly positive at the 1% level (0.8035***) Indirect effects are insignificant, while the overall effect is significantly positive at the 5% level (0.7017**). This indicates that the digital economy in the central region centers on local industrial clusters, with rapid development in e-commerce and smart manufacturing directly driving local efficiency gains. However, data circulation barriers and regional infrastructure gaps limit cross-regional spillovers, resulting in overall effects primarily driven by local positive factors.

In Western China, the direct effect of industrial intelligence is insignificant and negative, while the indirect effect is significantly positive at the 5% level (0.2042**). The total effect is significantly positive at the 10% level (0.1724*), exhibiting a typical “spillover-driven” pattern: Western regions possess weak industrial foundations, with late adoption and insufficient investment in intelligent manufacturing, resulting in weak local driving effects and even short-term negative impacts. However, its promotional role primarily stems from technological and industrial spillovers from eastern and central regions. The gradient transfer of intelligent manufacturing from the east and the diffusion of intelligent transformation experiences from the central region jointly drive improvements in carbon productivity in the west. The direct effect of the digital economy in the west is insignificant, while the indirect effect is extremely strong and positive at the 1% level (1.6849***), with the overall effect significantly positive at the 1% level (1.8286***). This reflects the western region’s high dependence on digital services, platform economy, and green technology spillovers from the eastern and central regions. Weak digital infrastructure and industrial foundations limit local pull effects, but cross-regional digital factor flows generate positive spillovers far exceeding local impacts, becoming the core driver of its green transformation.

Overall, the regional heterogeneity in industrial intelligence and the digital economy underscores the necessity of differentiated policies: The eastern regions should use fiscal, tax, and financial tools to alleviate the growing pains of intelligent transformation while strengthening cross-regional coordination in the digital economy. The central regions should dismantle administrative barriers and establish technology-sharing platforms to unlock spatial spillover potential. The western regions, meanwhile, should leverage regional collaboration mechanisms to proactively absorb digital and intelligent technology spillovers, accelerate infrastructure development, and advance green and low-carbon development through regional synergy.

## Conclusions and recommendations

This paper explores the impacts of industrial intelligence and the digital economy on regional TFCP and their spatial characteristics, and explores the regional heterogeneity. The conclusions as follows:

China’s regional TFCP grew steadily during the study period, but the “higher in the east and lower in the west” pattern persisted with a widening gap; the significantly positive global Moran’s I index of TFCP in all years confirmed its positive spatial autocorrelation, verifying the rationality of incorporating spatial factors into the research framework. Industrial intelligence and the digital economy are core drivers of regional TFCP improvement with distinct action mechanisms and spatial spillover features. The digital economy exerts significantly positive direct and indirect effects on TFCP, directly boosting local TFCP and driving that of neighboring areas through technology diffusion, industrial chain linkage and factor flow, forming a universal positive spatial radiation effect. Industrial intelligence has an insignificantly negative direct effect on local TFCP due to short-term resource misallocation, cost pressure and technology adaptation lag in early intelligent transformation, while its indirect effect is significantly positive at the 1% level; the total effect is significantly positive as positive spillover far outweighs the weak negative direct impact, reflecting its spatial externality with low-carbon dividends embodied more in cross-regional coordination. The two factors exhibit remarkable regional heterogeneity in their impacts on TFCP. For industrial intelligence, the eastern region shows a significantly negative direct effect and positive indirect effect with offset total effect due to early transformation adjustment costs; the central region has a significantly positive direct effect but insignificant indirect effect owing to an imperfect cross-regional technology diffusion mechanism; the western region presents an insignificant direct effect, with TFCP improvement driven by technological and industrial spillovers from the east and central regions in a typical spillover-driven pattern. For the digital economy, the eastern region forms a balanced “local driving + spatial spillover” dual-drive pattern supported by sound digital infrastructure and smooth factor flow; the central region has a significantly positive direct effect but insignificant indirect effect restricted by data barriers and infrastructure gaps; the western region’s TFCP improvement relies on substantial positive spatial spillovers from the east and central regions due to weak local digital driving capacity. Among control variables, coal-based energy consumption structure and secondary industry-dominated industrial structure exert prominent inhibitory effects on regional TFCP with strong negative spatial spillovers, reflecting obvious “regional linkage pollution” in China’s economic development; green finance’s positive potential to promote TFCP remains untapped due to late development, policy implementation lag and unbalanced regional resource allocation; FDI has an insignificantly positive direct effect and significantly negative indirect effect, as its induced spatial transfer of high-energy-consuming and high-emission industries inhibits neighboring TFCP, failing to form regional green synergy.

In summary, the development of TFCP needs to focus more on regional coordination between the digital economy and industrial intelligence, optimize energy and industrial structures, strengthen green financial support, and implement differentiated policies to promote balanced regional development. The following are the specific policy recommendations:

Promote the innovation of digital technology in an orderly manner. First, for cities with relatively weak digital policy support, governments should encourage digital enterprises to set up operations through diversified fiscal and tax policies and digital infrastructure development, and thereby stimulate the digital technology innovation vitality of the city. Second, with regard to non-resource-based cities, local governments should encourage digital technology innovations that are compatible with local industrial development and promote low-carbon growth, creating endogenous impetus for low-carbon development. Finally, in areas with weaker environmental regulations, environmental regulations should act as a key driver to encourage the clean digital technology innovation in order to strengthen the leading role of digital innovation in urban low-carbon development^42, 43^.

Implement differentiated industrial intelligence advancement with measure strategies. Dealing with regional heterogeneity of industrial intelligence impacts calls for a zoned guidance approach. Western regions should be priority areas for improving industrial intelligence with strong direct effects and positive spill-over effects through improved policy and financial support to achieve “overtaking on the curve” in emission reduction and efficiency gains. Central regions should focus on coordinating the intelligent upgrades and industrial transfer to enhance direct promotion effects. Eastern areas should focus on high-end breakthroughs and innovative R&D in industrial intelligence, and offer replicable advanced experiences for the country.

Accelerate the development of smart infrastructure, steadily advance the development of industrial intelligence, and make use of the “green effect” of smart technologies. The government focuses on creating smart demonstration areas of intelligent production, manufacturing, and selling, promoting speeding up the construction of smart infrastructure such as industrial Internet of Things (IoT) and 5G, to ensure that enterprises can fully unleash the intelligence of emission reduction capabilities. Enterprises strengthen independent innovation and R&D, establish smart equipment and workshops according to national standards to optimize production processes and reasonable allocation of resources. This meets requirements on high-efficiency production, reduced consumption and carbon emissions, improved quality, and cost reduction, respectively, to promote the development of low-carbon and energy-efficient models^44, 45^.

Establish incentive and compensation mechanisms on the basis of regional coordination to reduce the “siphon effect.” To solve the negative spatial spillovers (siphon effect) of industrial intelligence in some areas, cross-regional ecological compensation and coordinated development mechanisms should be developed. Through fiscal transfers, cross-regional project collaborations, and carbon emissions trading, make up to regions that are hurt by the spillover of technology or resource loss. Incentivize advanced areas to motivate lagging areas, turning “zero-sum games” into “win-win cooperation.” This ensures overall TFCP gains and equitable regional development.

## Data Availability

The datasets generated during and/or analysed during the current study are available from the corresponding author on reasonable request. All scripts and custom analysis code are deposited in an established DOI‑minting version control repository at [https://zenodo.org/](https:/zenodo.org), the DOI is: doi.org/10.5281/zenodo.18973539.
